# Hate and harassment in academia: the rising concern of the online environment

**DOI:** 10.1007/s10734-021-00787-4

**Published:** 2021-11-23

**Authors:** Atte Oksanen, Magdalena Celuch, Rita Latikka, Reetta Oksa, Nina Savela

**Affiliations:** Faculty of Social Sciences, Tampere University, Tampere, Finland

**Keywords:** Online environment, Social media, Internet, Online harassment, Cyberharassment, Cyberhate, Cyberbullying, Media, Academia, Occupational well-being

## Abstract

Hostile online communication is a global concern. Academic research and teaching staff are among those professionals who routinely give public comments and are thus vulnerable to online attacks. This social psychological and criminological study investigated online harassment victimization among university researchers and teachers. Survey participants (*N* = 2,492) were university research and teaching staff members from five major universities in Finland. Victimization was assessed with a 20-item inventory. The study included a wide range of both background and general measures on well-being at work. Participants also took part in an online experiment involving a death threat targeting a colleague. Results showed that 30% of the participants reported being victims of online harassment during the prior 6 months. Victims were more often senior staff members, minority group members, and from the social sciences and humanities. Those active in traditional or social media were much more likely to be targeted. Victims reported higher psychological distress, lower generalized trust, and lower perceived social support at work than non-victims. Individuals who were targeted by a colleague from their work community reported higher post-traumatic stress disorder scores and a higher impact of perceived online harassment on their work compared to other victims. In the experimental part of the study, participants reported more anxiety when a close colleague received a death threat. Participants also recommended more countermeasures to a close colleague than to an unknown person from the same research field. Results indicate that online harassment compromises well-being at work in academia. There is an urgent need to find ways of preventing online harassment, both in workplaces and in society at large.

## Introduction

Hostile online communication is a growing global concern (Keipi et al., 2017; Reichelmann et al., 2020; Williams, 2021). Online harassment (i.e., cyberharassment) covers a wide range of intensive and hostile actions that target individuals or groups (Farley et al., 2021; Näsi et al., 2017; Nurse, 2019). These types of negative actions are also analyzed with the concepts of online hate (i.e., cyberhate; online hate speech) and online bullying (i.e., cyberbullying). Online hate is a special category of online harassment that involves targeting either individuals or groups of people with intensive and hostile statements and content, such as insults concerning sexual orientation, ethnic background, or appearance (Hawdon et al., 2017). Online bullying also overlaps with harassment, and it has typically been investigated in the context of school or work (Kowalski et al., 2018; Oksanen et al., 2020a; Zych et al., 2015). This article uses the term “online harassment” to cover a large range of attacks on individuals via written or graphic insults, picture and video manipulations, identity thefts, and violent threats, well-known phenomena in online harassment literature (Bocij & McFarlane, 2003; Durkin & Patterson, 2012; Keipi et al., 2017; Näsi et al., 2017). It should be noted that perpetrators’ tools have multiplied in recent years. For instance, deep fake videos may constitute a major potential threat for any public discussion in the years to come (Botha & Pieterse, 2020; Lazer et al., 2018; van der Linden et al., 2020).

Thus far, much of the research on online harassment, hate, and bullying has focused on children, adolescents, and young adults (Hawdon et al., 2017; Keipi et al., 2017; Kowalski et al., 2014; Wachs et al., 2021a, b; Zych et al., 2015). By contrast, there is a lack of studies investigating the adult population in this context (Farley et al., 2021; Faucher et al., 2015; Jenaro et al., 2018; Kowalski et al., 2014; Oksanen et al., 2020a; Pew Research Center, 2021). Further, studies on academic staff facing such threats are scarce (Blizard, 2016; Cassidy et al., 2016, 2017; Gosse et al., 2021; Hodson et al., 2018; Jane, 2018; Veletsianos et al., 2018).

Academic researchers and teachers are a professional group potentially vulnerable to online harassment for multiple reasons. In addition to institutional hierarchy, academia includes other power imbalances caused by, for example, academic successes or mentoring relationships. Such imbalances create a fitting environment for issues regarding envy, heresy, and abuses of power (DeSouza, 2011). Fierce competition within the academic community can have negative side effects as well. Indeed, past research indicates that offline bullying is common in university settings (DeSouza, 2011; Giorgi, 2012; Keashly & Neuman, 2010). Attacked scholars have shared accounts of offline microaggressions and bullying, followed by inadequate responses from their institutions (Pyke, 2018). Due to the competitive nature of academia, the costs of reporting or intervening are often considered too high, which then upholds malpractice.

Public engagement is now a vital part of many researchers’ professional lives, including substantial interactions in online environments (Davies, 2013). Researchers use academic social networking sites for many purposes, including self-promotion, networking, collaboration, and knowledge sharing (Yan & Zang, 2019). Social networking sites are also used to communicate with students and foster their engagement (Dyson et al., 2015; Sheeran & Cummings, 2018). However, such visibility leaves academics vulnerable to attacks from strangers, as having a profession that requires an online presence has been linked to online victimization (Pew Research Center, 2014). Lately, the COVID-19 pandemic has forced university research and teaching staff to move much of their professional lives online, a shift leading to many new challenges (Watermeyer et al., 2021) while further exposing professionals to potential online harassment.

This is the first study to investigate online harassment among university researchers and teachers that utilizes an experimental design and a novel approach to identify the predictors of victimization. We analyze the reasons for and consequences of victimization as well as how people react when others are targeted.

### Risk factors and consequences for victims

Investigations of cybervictims have been theoretically grounded in social psychology and criminology (Keipi et al., 2017). Routine activities theory (RAT) is a major theory explaining the basic factors associated with victimization. According to RAT, crime takes place in a situation where a motivated offender and a suitable target are present, with a simultaneous lack of capable guardians (Cohen & Felson, 1979). Notably, such situations are very likely to happen in the online environment, where targets and offenders function in a shared space and guardianship is limited (Keipi et al., 2017). Although RAT was originally developed to describe the offline context, studies have successfully applied RAT to the online sphere (Hawdon et al., 2017; Holt & Bossler, 2008; Näsi et al., 2017; Wachs et al., 2021a, b). The online application of RAT takes into account that victims and offenders might share the same cyberspace without being present at exactly the same time; for example, a victimized person on Twitter might see an offending message much later (Reyns et al., 2011). Despite this asynchrony, cybervictimization has severe consequences for victims (Keipi et al., 2017).

RAT focuses on risk factors of victimization and states that individuals’ everyday routines can put them at risk of becoming targets of crimes by exposing them to dangerous circumstances. In the online context, a target’s visibility is one of the most crucial factors (Choi & Lee, 2017; Mikkola et al., 2021; Näsi et al., 2017), and for that reason, it is part of our focus in this article as well. Harassment may take place anywhere online, but social media sites are the most common places where such victimization occurs (Pew Research Center, 2021). Social media in this context refers to a wide variety of highly popular online platforms such as Facebook, Instagram, Twitter, and YouTube. In contrast to traditional media (e.g., television, radio, and newspapers), the content of social media is often disseminated by participants themselves. Social media thus makes it very easy to produce content and communicate with others (Keipi et al., 2017). On the basis of RAT, activity in the public sphere, via either traditional or social media, makes people potentially more exposed to offenders. According to the previous studies, the more visible potential victims are on different social media platforms, the more likely they are to be targeted (Keipi et al., 2017; Pew Research Center, 2021). Having a job that requires an online presence, or a job where the internet is an essential tool, as is the case for many researchers, is also a significant predictor of becoming a target (Kowalski et al., 2012).

In the general population, young women are especially likely to become targets of some of the most severe instances of online harassment (Pew Research Center, 2021). Also, people with minority status have been targeted based on their physical appearance and sexual orientation (Costello et al., 2019; Gosse et al., 2021). Previous studies have shown that online harassment against academics often targets minority members and women (Barlow & Awan, 2016; Cassidy et al., 2016; Kavanagh & Brown, 2020; Nagle, 2018; Veletsianos et al., 2018; Yelin & Clancy, 2020).

Personality traits are important in understanding online harassment as a phenomenon. The Big Five model is one of the most common and widely accepted taxonomies of human personality (Digman, 1990; John et al., 2008). Previous research has found high neuroticism, extroversion, and openness, as well as low agreeableness and conscientiousness to be associated with cybervictimization (ElSherief et al., 2018; Kowalski et al., 2012; Peluchette et al., 2015). Impulsivity has also been tied to offline and online victimization among adolescents and young adults (Álvarez-García et al., 2019; Bossler & Holt, 2010; Fanti et al., 2012; Kokkinos et al., 2014; López-Larrañaga & Orue, 2019; Mikkola et al., 2021; Pratt et al., 2014; Vazsonyi et al., 2012) and among the adult population (Nedelec, 2018). Impulsivity is related to co-occurring victimization both offline and online (Nedelec, 2018). Impulsive individuals are more likely to offend others (Fanti et al., 2012; Kokkinos et al., 2014; Vazsonyi et al., 2012; Zych et al., 2021) and less likely to help victims as bystanders (Erreygers et al., 2016).

Past research indicates that cyberharassed scholars may suffer a wide range of negative consequences, including various negative emotions, physical signs of stress, sleep problems, difficulty concentrating, post-traumatic stress disorder (PTSD) symptoms, suicidal thoughts, and long-lasting mental health effects such as anxiety and depression (Blizard, 2016; Cassidy et al., 2016, 2017). Cyberharassment can negatively influence scholars’ professional lives, causing a loss of productivity and self-confidence, thoughts of quitting one’s job, and lowered job satisfaction (Blizard, 2016; Cassidy et al., 2016, 2017; Coyne et al., 2017; Gosse et al., 2021).

Victims of attacks in academia often seek social support from colleagues, friends, family, supervisors, or therapists (Blizard, 2016; Cassidy et al., 2016, 2017). They use a wide range of coping strategies, usually implementing multiple methods at once, especially in cases of severe attacks (Veletsianos et al., 2018). However, online offences are often underreported, with studies suggesting that many, or even the majority, of targeted individuals do not report the event to any authorities (Blizard, 2016; Cassidy et al., 2016, 2017; Faucher et al., 2015). Reasons for not reporting to the police are tied to a lack of trust in police and the limited capability of law enforcement to solve these issues due to their complex nature and lack of necessary resources and expertise (Holt & Lee, 2019; Koziarski & Lee, 2020).

### Social identity theory approach to bystander reactions

In social media, messages stay online for a prolonged time and may be visible to large groups of users. Because of this, online harassment is likely to involve bystanders who directly witness offensive acts. Thus, online attacks also have a potential impact on other people, for example, colleagues and family members of those who have been targeted (D’Cruz & Noronha, 2013). For these reasons, it is highly important to understand how people in general react to online harassment. Previous studies on bystanders have shown that they are more supportive when the target is closer to them (Dickter, 2012; Machackova et al., 2013). Also, perceived psychological closeness increases prosocial behavior (Korchmaros & Kenny, 2001; Lee & Kim, 2020; Passarelli & Buchanan, 2020). That is, if someone sees a close friend or family member in danger, they are more likely to help them or feel concerned.

The social identity theory (SIT) framework that was first introduced by Tajfel and Turner (1979) helps us to further understand how people react to online harassment. SIT proposes that people will form consequential group identities on a minimal basis (Abrams & Hogg, 2006; Brown, 2000; Tajfel & Turner, 1979; Turner et al., 1987). Individuals are then motivated to view their own in-group in a positive light (Turner et al., 1979) and can experience emotions associated with self-categorizing as a member of a social group (Smith & Mackie, 2015; Turner et al., 1987).

In accordance with SIT predictions, studies have shown that people feel more empathy toward in-group than outgroup members (Cikara et al., 2011; Vanman, 2016). Empathy may, in turn, drive bystanders to help cyberbullying victims (Erreygers et al., 2016; Freis & Gurung, 2013; Kowalski et al., 2014; Lambe et al., 2019; Machackova & Pfetsch, 2016; Van Cleemput et al., 2014). Empathizing with the victim generates a co-victimization experience and causes stress, which may motivate individuals to defend the victim (Coyne et al., 2019). Importantly, feeling empathy can be a target-dependent state. For instance, individuals are motivated to feel more empathy toward people who share their political views (Hasson et al., 2018). Like-mindedness has been argued to be a stand-alone reason for feeling empathy toward an individual (Stevens et al., 2020).

Based on these perspectives, we expected that both closeness to and like-mindedness with the victim explain how people react when witnessing online harassment. Feeling concerned for closely connected or similarly minded others is easy due to group membership, and it may facilitate reacting against online harassment and supporting the victims. In addition, both closeness and like-mindedness are important factors of social identity that can be threatened if other in-group members are targeted, making it more necessary for bystanders to react.

### This study

Our aim was to investigate online harassment among research and teaching staff. The study adds to the discussion within higher education studies on public engagement and the use of social media by academic staff. This study is grounded in theoretical paradigms of cybervictimization and both criminological and social psychological theories that help to understand why and how academic staff are victimized, what the general consequences are of hate and harassment, and potential reactions toward it.

The first part of our analysis investigated the risk and protective factors of online harassment victimization. Our main hypothesis was that both traditional media and social media presence are associated with online harassment victimization (H1). This hypothesis was based on empirical research on RAT showing that visibility of the potential victim increases the likelihood of victimization. In other words, individuals who are often present in traditional and social media become targeted. Our analysis considered other risk factors, including background factors and personality, but exact hypotheses were not made due to the lack of previous studies among research staff.

The second part of the analysis focused on the potential consequences of online harassment on well-being and professional life. Our main hypothesis was that online harassment victims differ from non-victims (H2). This hypothesis was generally based on previous studies on online victimization.

The third part of the analysis was based on the experimental design investigating reactions to online harassment. Within our experiment, we investigated whether reactions to a death threat were more severe when the victim was close to or like-minded with the subject. Our hypotheses were preregistered to Open Science Forum (Oksanen et al., 2020b). We expected that closeness to the victim would increase both state anxiety (H3a) and the number of suggested countermeasures (H3b). We also expected that like-mindedness with the victim increased both state anxiety (H4a) and the number of suggested countermeasures (H4b).

## Method

### Participants and procedure

Participants (*N* = 2,492) of this study were university research and teaching staff members from five major universities in Finland that were geographically diverse, representing major universities in the southern, western, eastern, and northern parts of the country. Participants were 53.17% female, 46.31% male, and 0.52% other gender, and the mean age was 43.24 years (*SD* = 11.21). Participants were sampled based on contact information received from HR departments and university websites. The random sample included half of the university staff from each university. All participants were contacted by email, and the data was collected between 15 April and 5 June 2020. The overall response rate was 50.06%, and 40.79% finished the survey. Participants who completed the full survey were included in this study. There was no major bias due to the non-response based on the analysis of age and gender distributions. There were slightly more female participants compared to investigated universities (53.17% vs. 48.05%), but the mean age was almost the same (43.24 vs. 43.43 years).

Participants were asked about their experiences with online harassment due to their work. Those who reported victimization during the prior 6 months were asked where the acts took place, whether they knew the perpetrator, whether they reported the actions to someone, and what the consequences of the victimization were. In addition, the survey included background information and measures on personality and well-being. Median response time to the survey was 14:45 min. The survey was conducted in both Finnish and English.

The survey also included a survey experiment that was preregistered to Open Science Forum (Oksanen et al., 2020b). Participants were randomly assigned to one of four different groups and asked to imagine a situation where someone had received a personal death threat on social media after giving a public interview. This between-subject experiment manipulated (a) closeness to the victim (close colleague/unknown person from the same research field) and (b) like-mindedness (agrees with the interview statements/disagrees with the interview statements). Anxiety and countermeasure reactions were asked of the participants after this. These are explained in the “Measures” section.

Participation was voluntary, and the participants were informed about the study’s aims. They were assured that they could stop at any time and delete their information when answering to the survey. The research group administered the data collection. The academic ethics committee of Tampere region in Finland stated (decision 17/2020) that the study protocol did not include any ethical concerns.

### Measures

#### Online harassment victimization

We measured whether participants had been victims of online harassment during the prior 6 months following situations at their work or because of their work. Participants filled out a 20-item inventory on different forms of online hate and harassment, from personal insults (e.g., “attacks against you as a person, your values, or your personal life have been made”) to violent threats (e.g., “you have been threatened with violence”) (see Appendix Table 5 for the full list). The response options ranged from “never” to “daily.” Some inventory items were based on items of a “cyberbullying at work” survey (Forssell, 2016; Oksanen et al., 2020a), and others were from studies investigating online harassment and hate in general (Keipi et al., 2017; Reichelmann et al., 2020).

#### Background factors

Background factors included gender and age. Participants were also asked whether they considered themselves a member of a minority group, whether they had a PhD degree, a long-term contract, and whether they were in a managerial position. The scientific field was assessed with a longer list of questions but categorized into five major groups: natural sciences, engineering and technology, medical and health sciences, humanities, and social sciences.

#### Target availability

We based target availability measures on RAT and cybervictimization research (Holt & Bossler, 2008; Näsi et al., 2017; Williams, 2016). Measures included media appearances asked with the question: “How often are you on TV, radio, or in the newspaper because of your work*?*” A dummy variable was created indicating those who had media appearances at least monthly (0 = no, 1 = yes). We also asked about how often they wrote messages or posted content to the most popular social media sites. A dummy variable was created indicating those who wrote messages or posted content to Facebook, Twitter, Instagram, YouTube, LinkedIn, blogs, or discussion forums on a weekly basis (0 = no, 1 = yes). Victimization by other means was asked with the question: “Have you received abusive or threatening messages through other means than via the internet and social media?” Those who selected victimization via telephone, messages (e.g., text messages), mail, or face-to-face were categorized as victims (0 = no victimization by other means, 1 = victimization by other means). In addition, we asked whether participants had attacked others with the question: “During the past 6 months, how often have you sent abusive or threatening messages to other people?” Those selecting sometimes or more often were categorized as offending others (0 = does not offend others, 1 = has offended others).

#### Personality

We measured personality traits with a 15-item Big Five inventory (Hahn et al., 2012). Each item had response options from 1 (*strongly disagree*) to 7 (*strongly agree*). For each personality trait, we created a 3-item sum variable ranging from 3 to 21. Inter-item reliability based on McDonald’s omega ranged from acceptable to good: openness (ω = 0.68), conscientiousness (ω = 0.55), extroversion (ω = 0.85), agreeableness (ω = 0.61), and neuroticism (ω = 0.79). Impulsivity was measured with the Eysenck Impulsiveness Scale (Eysenck & Eysenck, 1978). Response options were no (0) and yes (1) for all questions. This measure was recently used and validated in other cybervictimization research (Mikkola et al., 2021; Zych et al., 2021). It showed an acceptable inter-item reliability: ω = 0.72. The scale ranged from 0 to 5 (*M* = 0.95, *SD* = 1.32), with higher scores indicating higher impulsiveness.

#### Well-being

Our study included several established measures aimed at estimating the general well-being of the participants at work. Work engagement was measured with the Utrech Work Engagement Scale (UWES-9) (Schaufeli & Bakker, 2004). The measure showed excellent inter-item reliability: ω = 0.94. The scale ranged from 0 to 54 (*M* = 42.31, *SD* = 9.53), with higher scores indicating higher work engagement. Psychological distress was measured with a 12-item general health questionnaire (GHQ-12; Goldberg et al., 1997). The measure showed excellent inter-item reliability: ω = 0.87. Likert coding (0–1–2–3) was applied, and the scale ranged from 0 to 36 (*M* = 13.08, *SD* = 5.51), with higher scores indicating higher psychological distress. Self-esteem was measured with a single-item self-esteem scale including a statement: “I have high self-esteem” (Robins et al., 2001). The scale ranged from 1 (*not very true of me*) to 7 (*very true of me*; *M* = 5.00, *SD* = 1.40). Generalized trust was measured with the standardized single-item measure: “Generally speaking, would you say that most people can be trusted, or that you can’t be too careful in dealing with people?” (Delhey & Newton, 2005; Uslaner, 2002). The response scale ranged from 1 (*Need to be very careful in dealing with people*) to 7 (*Most people can be trusted*; *M* = 5.10, *SD* = 1.59). Perceived social support at work was assessed with a 3-item scale that asked about help and support gained from colleagues, closest supervisor, and employer. All items had response options ranging from 1 (*not at all likely*) to 7 (*very likely*). The scale had a good inter-item reliability, ω = 0.79, and a range from 3 to 21 (*M* = 14.97, *SD* = 4.36), with higher scores indicating perceived support at work. All participants were asked a single-item measure about the perceived impact of online harassment on work: “Has the generalization of hostile, harassing, or threatening speech affected your actions as a professional in your own field?” The scale ranged from 1 (*not at all*) to 7 (*very much so*; *M* = 1.90, *SD* = 1.41). Negative impacts of online harassment on the victims were assessed with a trauma screening questionnaire designed to screen PTSD (Brewin et al., 2002). In our adapted 10-item scale, we asked participants to report symptoms occurring at least twice during the past week. The scale had a good inter-item reliability, ω = 0.76, and a range from 0 to 10 (*M* = 0.90, *SD* = 1.57).

#### Experiment

A six-item short-form of the state scale of the Spielberger State—Trait Anxiety Inventory (STAI-6, Marteau & Bekker, 1992) was adapted to measure perceived state anxiety in the experiment after reading the hypothetical scenario on death threats. On average, the inter-item reliability of the scale was good in all scenarios (*M* ω = 0.84; range from 0.81 to 0.85). The scale had a range from 6 to 42. Participants were also asked several questions about the 10 countermeasures they would suggest to the victim, for example, blocking the perpetrator, turning to counseling, and reporting the incident to the police (see Appendix 2 for the full list). Table 1 reports the exact wording given for each experimental group and the means of their anxiety and reaction scores.

**Table 1:** Descriptive overview of four experimental groups (*n* = 2,490)

	Group 1 (close & like-minded victim)	Group 2 (close & differently minded victim)	Group 3 (not close & like-minded victim	Group 4 (not close & differently minded victim
Wording	Think about a situation in which your **close colleague** gives a public interview. You **completely agree** with the views given in the interview. After the interview he/she will get a death threat personally on social media	Think about a situation in which your **close colleague** gives a public interview. You **completely disagree** with the views given in the interview. After the interview he/she will get a death threat personally on social media	Think about a situation in which **an unknown person from your field** gives a public interview. You **completely agree** with the views given in the interview. After the interview he/she will get a death threat personally on social media	Think about a situation in which **an unknown person from your field** gives a public interview. You **completely disagree** with the views given in the interview. After the interview he/she will get a death threat personally on social media
*n*	593	664	612	621
*M* (SD) anxiety	30.97 (7.57)	31.53 (7.31)	28.64 (7.65)	28.88 (7.12)
*M* (SD) suggested countermeasures	2.33 (1.12)	2.42 (1.21)	2.20 (1.03)	2.23 (1.10)

### Statistical analyses

We conducted all analyses with Stata 16. Descriptive methods were used to provide the data’s general characteristics and to investigate differences between victims and non-victims and different types of victims. Statistical significances were tested with the chi squared test (χ^2^) for categorical variables and the 2-tailed *t*-test for continuous variables. We also reported effect sizes with Cramér’s V for categorical variables and Cohen’s d for continuous variables.

Regression models further investigated the associations. Our first goal was to analyze with logistic regression those who were victims of online harassment. We reported odd rations (OR), their 95% confidence intervals, average marginal effects, and *p*-values for statistical significance. Average marginal effects (AME) indicate how much the likelihood of the investigated phenomenon increases when the independent variable increases by one unit. AME coefficients are hence often more concrete than standard odds ratios and are more reliable when comparing results from different logistic models (Mood, 2010). Model statistics included pseudo coefficients of determination (Cragg-Uhler/Nagelkerke pseudo *R*^2^). Assumptions of logistic regression were met, and we did not find issues with multicollinearity.

Ordinary least squares regression was used for the analysis of work engagement, psychological distress, self-esteem, generalized trust, perceived social support, and perceived impact of online harassment on work. These were all used separately as dependent variables. We first investigated the differences between victims and non-victims (*n* = 2479) and then differences between different types of victims (*n* = 744). All models were adjusted for gender, age, scientific field, education, contract type, managerial position, and minority status. For model estimation reasons, those identifying as other gender were dropped from the regression models. We reported unstandardized regression coefficients (B) and their robust Huber–White standard errors (B SE), standardized beta coefficients (β), and *p*-values. Robust standard errors were used due to the heteroskedasticity of residuals. We also tested other assumptions of OLS and found no issues that could cause bias to the results.

For analyzing the experiment’s results, we used analysis of variances (ANOVA). Bartlett’s test for equality of variances showed no issues with manipulated factors in state anxiety. In terms of the analysis of suggested countermeasures, issues were found with unequal variance. We solved the issue with square root transformation of the suggested countermeasures variable. Our sample size was large (*N* = 2,492), and for this reason, we were not concerned with minor violations to normality in general. With larger sample sizes, the issue of assumption of normality is not considered crucial (Tabachnick & Fidell, 2013; Waternaux, 1976). All experimental groups were relatively equal in size. We reported the degrees of freedom (df), mean squares (MS), *F*-test value (F), statistical significance (*p*), and partial eta-squared (η^2^_p_).

## Results

### Characteristics of online harassment victims

Among university research and teaching staff participants (*N* = 2,492), 30.06% (*n* = 749) reported exposure to online harassment at least sometimes during the prior 6 months. The prevalence of victimization ranged from 27.09 to 31.84% in five universities, but the differences between universities were not statistically significant. The percentage of those reporting monthly victimization was 5.18%, and 1.18% reported weekly victimization during the investigated period.

The most common forms of online harassment involved excessive criticism going beyond normal critique (16.69%), receiving offending and angry messages (15.97%), and attacks against participants as persons (13.76%). Notably, relatively many reported clearer cut victimization to things such as identity theft (1.36%), violent threat (2.81%), and death threat (0.96%). Those victimized (*n* = 749) reported, on average, victimization to four asked categories (*M* = 3.89, *SD* = 2.94).

Women reported more commonly than men that they were underestimated or criticized online because of their gender (13.51% vs. 5.20%, χ^2^ = 48.90, *p* < 0.001) and that they were sexually harassed online (8.00% vs. 2.69%, χ^2^ = 33.36, *p* < 0.001). Men reported more often than women that they were attacked online because of their religion or ideology (7.97% vs. 5.13%, χ^2^ = 8.24, *p* = 0.004).

Out of the victims of online harassment, 42.99% knew the perpetrators, 33.11% did not know the perpetrators (but they were using their own names), and 23.90% said that offenders used pseudonyms or were otherwise unknown to the participant. In 17.76% (*n* = 133) of the cases, the perpetrator was a member of the respondent’s work community at their university.

Out of the victims, only 16.29% reported the event to their supervisors and 3.34% to the police. We asked about several reasons why the victims did not report the acts to the police: 76.10% of them considered that the act was not serious enough. In addition, out of victims 22.79% stated that they did not believe anything would have been done to the case.

It is remarkable that almost half (45.95%) of those who had been threatened with violence considered the act to be not serious enough to report it to police. The same is valid for victims of other types of online harassment. For example, 47.06% of identity theft victims considered the act not serious enough to report it to police.

### Comparison of victims and non-victims

Comparisons of victims and non-victims are portrayed in Table 2. The results showed that there were no statistically significant differences between genders. Age was only significant in the descriptive findings (χ^2^[2] = 29.77, *p* < 0.001, V = 0.11), but not in the full logistic model (Cragg-Uhler pseudo-R^2^ = 0.203). Those with minority identity status reported more victimization in the descriptive findings (χ^2^[2] = 14.95, *p* < 0.001, *V* = 0.08) and in the full logistic model (OR = 1.55, AME = 0.078, *p* = 0.001). Descriptive findings also showed differences between scientific fields (χ^2^[4] = 46.32, *p* < 0.001, V = 0.14). Victimization was particularly high among social scientists and humanists. Of the social scientists, 37% reported victimization. In the full model adjusting the number of factors, they reported on average 9% higher victimization than the reference group of natural scientists (AME = 0.087, *p* < 0.001). Those having a PhD degree reported higher victimization than others in both the descriptive analysis (χ^2^[1] = 41.11, *p* < 0.001, *V* = 0.13) and logistic model (OR = 1.57, AME = 0.079, *p* < 0.001). Those in managerial positions reported higher victimization in both the descriptive analysis (χ^2^[1] = 35.13, *p* < 0.001, V = 0.12) and logistic model (OR = 1.41, AME = 0.060, *p* = 0.006).

**Table 2:** Descriptive statistics and logistic regression model on online harassment victimization among university research and teaching staff in Finland (*N* = 2,492)

	Victim					Logistic model				
	*n* (no)	% (no)	*n* (yes)	% yes)	*p*	OR	95%	CI	AME	*p*
Background										
Gender					0.190					
Male	827	71.66	327	28.34		*ref*	*ref*	*ref*	*ref*	*ref*
Female	908	68.53	417	31.47		1.03	0.83	1.27	0.005	0.798
Other	8	61.54	5	38.46		-	-	-	-	-
Age					< 0.001					
> 50	449	63.33	260	36.67		*ref*	*ref*	*ref*	*ref*	*ref*
31–50	1,025	70.98	419	29.02		0.91	0.61	1.37	-0.017	0.653
≤ 30	269	79.35	70	20.65		0.83	0.65	1.05	-0.033	0.125
Minority identity					< 0.001					
No	1,517	71.42	607	28.58		*ref*	*ref*	*ref*	*ref*	*ref*
Yes	226	61.41	142	38.59		1.55	1.20	2.00	0.078	0.001
Scientific field					< 0.001					
Natural sciences	578	77.69	166	22.31		*ref*	*ref*	*ref*	*ref*	*ref*
Engineering and tech	217	73.31	79	26.69		1.44	1.02	2.01	0.063	0.035
Medical & health sci	265	71.24	107	28.76		1.23	0.90	1.68	0.035	0.187
Humanities	251	63.87	142	36.13		1.50	1.10	2.03	0.070	0.009
Social sciences	432	62.88	255	37.12		1.63	1.26	2.12	0.087	< 0.001
PhD degree					< 0.001					
No	672	78.05	189	21.95		*ref*	*ref*	*ref*	*ref*	*ref*
Yes	1,071	65.67	560	34.33		1.57	1.22	2.02	0.079	< 0.001
Long-term contract					0.003					
No	1,045	72.27	401	27.73		*ref*	*ref*	*ref*	*ref*	*ref*
Yes	698	66.73	348	33.27		0.88	0.71	1.10	–0.022	0.274
Managerial position					< 0.001					
No	1,388	72.98	514	27.02		*ref*	*ref*	*ref*	*ref*	*ref*
Yes	355	60.17	235	39.83		1.41	1.10	1.79	0.060	0.006
Target availability										
Media appearances monthly					< 0.001					
No	1,697	72.03	659	27.97		*ref*	*ref*	*ref*	*ref*	*ref*
Yes	46	33.82	90	66.18		2.87	1.91	4.31	0.187	< 0.001
Weekly some postings					< 0.001					
No	1,208	77.29	355	22.71		*ref*	*ref*	*ref*	*ref*	*ref*
Yes	535	57.59	394	42.41		2.12	1.74	2.57	0.133	< 0.001
Victim by other means					< 0.001					
No	1,514	74.99	505	25.01		*ref*	*ref*	*ref*	*ref*	*ref*
Yes	229	48.41	244	51.59		2.64	2.10	3.32	0.172	< 0.001
Has offended others					< 0.001					
No	1,735	70.39	730	29.61		*ref*	*ref*	*ref*	*ref*	*ref*
Yes	8	29.63	19	70.37		5.71	2.22	14.74	0.309	< 0.001
Personality	M (no)	SD (no)	M (yes)	SD (yes)	*p*					
Openness	15.26	3.32	16.17	3.08	< 0.001	1.05	1.02	1.09	0.009	0.001
Conscientiousness	14.48	3.13	14.82	3.22	0.014	1.02	0.98	1.05	0.003	0.331
Extroversion	12.80	4.35	13.86	4.09	< 0.001	1.01	0.99	1.04	0.002	0.406
Agreeableness	15.27	2.96	14.96	3.06	0.015	0.96	0.93	1.00	− 0.006	0.032
Neuroticism	12.47	4.05	12.16	4.05	0.077	1.00	0.97	1.02	0.000	0.880
Impulsivity	0.85	1.24	1.18	1.46	< 0.001	1.14	1.06	1.23	0.023	< 0.001

Analysis of target availability factors in Table 2 shows that monthly media appearances and weekly social media postings were strongly associated with being a victim (*p* < 0.001) in both descriptive analyses and the full logistic model. For example, two-thirds of the participants who had monthly appearances in the media reported online harassment victimization, while less than a third of those who did not have monthly appearances in the media were targeted (χ^2^[1] = 89.28, *p* < 0.001, *V* = 0.19). They were also more likely to be victims than others based on the full model (OR = 2.87, AME = 0.187, *p* < 0.001). Similar effect sizes were found for weekly social media postings in the descriptive analyses (χ^2^[1] = 107.55, *p* < 0.001, *V* = 0.21) and full logistic model (OR = 2.12, AME = 0.133, *p* < 0.001). Victims of online harassment also reported significantly more victimization by other means, such as telephone or face-to-face, based on descriptive findings (χ^2^[1] = 128.72, *p* < 0.001, *V* = 0.23) and the full model (OR = 2.64, AME = 0.172, *p* < 0.001). Those participants who reported offending others (*n* = 27, 1.08% out of all participants) had a very high likelihood of personal victimization, and over 70% of them were victims (χ^2^[1] = 21.10, *p* < 0.001, *V* = 0.09). In the full adjusted model, they had on average a 31% higher rate of victimization than those not offending others (AME = 0.309, *p* < 0.001).

The analysis of personality in Table 2 shows evidence that impulsivity was linked to online harassment victimization in the descriptive analysis (*t*[2490] = 5.72, *p* < 0.001, *d* = 0.25). In the full logistic model, impulsivity was also associated with online harassment victimization (OR = 1.14, AME = 0.02, *p* < 0.001). Analysis of Big Five personality traits showed that higher openness (*t*[2490] = 6.35, *p* < 0.001, d = 0.28), higher conscientiousness (*t*[2490] = 2.47, *p* = 0.014, d = 0.11), higher extroversion (t[2490] = 5.69, *p* < 0.001, d = 0.25), and lower agreeableness (*t*[2490] = 2.43, *p* = 0.015, d = 0.11) were associated with online harassment victimization. Out of these, only higher openness (OR = 1.05, AME = 0.009, *p* = 0.001) and lower agreeableness (OR = 0.96, AME = 0.006, *p* = 0.032) were associated with online harassment victimization in the full model.

The comparison of victims, non-victims, and different types of victims is reported in Table 3. Descriptive analysis based on *t*-tests showed that victims of online harassment reported higher psychological distress (*t*[2490] = 2.52, *p* = 0.012, *d* = 0.11), higher self-esteem (*t*[2490] = 2.46, *p* = 0.014, *d* = 0.11), lower generalized trust (*t*[2490] = 2.91, *p* = 0.004, *d* = 0.13), and low perceived social support at work (*t*[2490] = 6.18, *p* < 0.001, *d* = 0.27). The victims believed that hostile, harassing, or threatening speech had affected their actions as professionals (*t*[2490] = 3.08, *p* = 0.002, *d* = 0.13). Regression models further investigated the differences between victims and non-victims while adjusting for gender, age, scientific field, education, contract type, supervisory position, and minority status. The results showed that victims of online harassment reported higher psychological distress (β = 0.07*, p* = 0.001), lower generalized trust (β =  − 0.08, *p* < 0.001), and low perceived social support at work (β =  − 0.11, *p* < 0.001).

**Table 3:** Comparison of victims and non-victims and different types of victims (*t*-test mean comparison and regression models)

All (*N* = 2,492)	Victim of harassment or hate			Effects for victims in models (*n* = 2,479)			
	No	Yes	*p*	B	SE (B)	β	*p*
Work engagement	42.19	42.61	0.316	− 0.43	0.43	− 0.02	0.315
Psychological distress	12.89	13.50	0.012	0.86	0.24	0.07	< 0.001
Self-esteem	4.96	5.11	0.014	0.06	0.06	0.02	0.334
Generalized trust	5.17	4.96	0.004	− 0.28	0.07	− 0.08	< 0.001
Perceived social support	15.32	14.15	< 0.001	− 1.01	0.20	− 0.11	< 0.001
Perceived impact of hate and harassment on work	1.84	2.03	0.002	0.11	0.06	0.04	0.091
Among victims (*n* = 749)	Perpetrator from the University community			Effects for victims in models (*n* = 744)			
	No	Yes	*p*	B	SE (B)	β	*p*
PTSD	0.73	1.71	< 0.001	0.95	0.19	0.23	< 0.001
Work engagement	42.99	40.85	0.024	− 2.31	1.02	− 0.09	0.024
Psychological distress	13.52	13.39	0.809	− 0.42	0.59	− 0.03	0.474
Self-esteem	5.14	4.94	0.134	− 0.16	0.15	− 0.04	0.275
Generalized trust	5.01	4.76	0.106	− 0.23	0.16	− 0.06	0.155
Perceived social support	14.37	13.12	0.005	− 1.29	0.47	− 0.11	0.006
Perceived impact of hate and harassment on work	1.91	2.57	< 0.001	0.62	0.17	0.16	< 0.001

Table 3 includes an analysis of online harassment victims whose cases had the perpetrators coming from outside and inside the work community. Those victims whose perpetrators came from the university community reported higher PTSD scores (*t*[2490] = 6.76, *p* < 0.001, *d* = 0.65), lower work engagement (*t*[2490] = 2.26, *p* = 0.024, *d* = 0.22), lower perceived social support (*t*[2490] = 2.81, *p* = 0.005, *d* = 0.27), and a higher perceived impact of online harassment on work (*t*[2490] = 4.73, *p* < 0.001, *d* = 0.45). The same factors remained statistically significant in the regression models adjusting for gender, age, scientific field, education, contract type, supervisory position, and minority status. The results showed that victims whose perpetrators came from the university community reported higher PTSD scores (β = 0.23*, p* < 0.001), lower work engagement (β =  − 0.09, *p* = 0.024), lower perceived social support at work (β =  − 0.11, *p* = 0.006), and higher perceived impact of online harassment on work (β = 0.16, *p* < 0.001).

### Reactions to death threat in the experiment

The last part of our analysis used an experiment involving a hypothetical death threat to another person. Participants were asked how anxious they felt about the situation and what kind of recommendations they would give to the person facing a death threat. Manipulated factors were closeness and like-mindedness to the victim. Descriptive results on the group differences are reported in Table 1 and show that groups 1 and 2 involving a close victim reported higher anxiety and a number of suggested countermeasures than groups 3 and 4 involving not being close to the victim. The one-way ANOVA results for the four groups showed statistically significant differences between groups on analysis of anxiety (F[3,2489] = 24.47, *p* < 0.001) and reactions (F[3,2489] = 4.39, *p* = 0.004). A pairwise comparison of means using Tukey’s honest significant difference test indicated that groups involving being close to the victim reported higher anxiety and number of countermeasures than groups involving not being close to the victim.

A two-way ANOVA was run to analyze the effect of closeness (close vs. not close) and like-mindedness (like-minded vs. non-like-minded). Results are reported in Table 4. Participants expressed higher anxiety when the victim was close to them (F[1,2486] = 70.35, *p* < 0.001), and they suggested more countermeasures (F[1,2486] = 10.57, *p* = 0.001). Like-mindedness did not have any effect in the analyses. The effect sizes (η_p_^2^) of the suggested countermeasures were below 0.01 and could be considered very weak.

**Table 4:** Two-way ANOVA of anxiety cause by the situation and recommendations given for the victim in a death threat experiment

	Anxiety					Recommendations				
Measure	df	MS	*F*	*p*	η_p_^2^	df	MS	*F*	*p*	η _p_^2^
Close	1	3865.48	70.35	< 0.001	0.028	1	1.87	10.57	0.001	0.005
Like-minded	1	98.79	1.80	0.180	0.001	1	0.14	0.8	0.371	0.000
Close X like-minded	2	16.23	0.30	0.587	0.000	1	0.24	1.36	0.244	0.001
Residual	2,486	54.94				2,486	0.18			
Total	2,489	56.50				2,489	0.18			

Recommendations variable was square root transformed

## Discussion

### Results overview

This study investigated online harassment victimization among university researchers and teachers. Results showed that 30% reported victimization to online harassment during the prior 6 months, with 5% reporting victimization on a monthly basis and 1% on a weekly basis. Victims were more often senior staff members, minority group members, and from social sciences and humanities. In over half of the cases, the perpetrator was a person unknown to the victim. Only 16% of the victims reported the attack to their supervisors and 3% to the police, although many had faced serious offences (e.g., violent threats).

Those active in traditional media or social media were victims significantly more often, which confirmed H1. This result is in line with previous empirical findings on the relationship between online activity and victimization (Choi & Lee, 2017; Keipi et al., 2017; Williams, 2016), previous research on visibility on social media platforms (Näsi et al., 2017; Pew Research Center, 2021), and job-related usage of the internet and online presence (Kowalski et al., 2012; Oksanen et al., 2020a) as determining factors of becoming a target.

Our results provided confirmation for H2 because victims reported higher psychological distress, lower trust, and lower perceived social support at work than non-victims. Victims who were targeted by a member of their university community reported higher PTSD symptoms and higher perceived impacts of online harassment on work than other victims. Previous studies on cyberbullying at work have indicated similar associations of detrimental effects to the victims (Blizard, 2016; Cassidy et al., 2017; Oksanen et al., 2020a).

Results based on our online experiment confirmed our preregistered hypotheses (H3a and H3b) because participants reported more anxiety and were more willing to suggest countermeasures when a close colleague faced a death threat rather than an unknown person from the same field. Hypotheses (H4a and H4b) on like-mindedness were not confirmed. Although group memberships, especially online, are often based on like-mindedness only, this is a weaker factor for group membership than directly indicated closeness. Overall, our results bring important new experimental evidence on bystanders’ reactions. Previously, bystanders of online hate and harassment have been studied only with correlational designs.

### Theoretical and practical implications

Our research was based on both criminological and social psychological theories. We believe the combination of these two is important in understanding online harassment and making the contribution meaningful to higher education studies. Social psychological theories on group behavior are especially crucial when analyzing online behavior. Our results indicate that closeness of others had an impact on how strongly people reacted to online harassment. This has practical implications in terms of how bystanders might react only to assaults against their close colleagues. Within social media platforms, such passivity might lead to further problems, because social media platforms have been quite ineffective in managing online aggression over the years. Our study expands both theoretically and empirically the current discussion in higher education studies on the social media usage and public engagement of academic staff. The online environment is rapidly changing in alarming directions, and focus should be placed on malicious activities directed against academics who may be facing increasing pressure to be active in social media. These demands are especially accentuated in light of the shift to remote work brought about by the COVID-19 pandemic.

Hostile, hateful, and harassing behavior has become a mainstream and highly common phenomenon on Facebook and Twitter, which has serious implications for democracy and freedom of speech (Hawdon et al., 2017; Keipi et al., 2017; Reichelmann et al., 2020). The European Commission (2020) recently proposed a legislative renewal for digital services that would put pressure on largest online platforms to take a more active stance against illegal content online. This is highly critical because social media users can only partially protect themselves from being attacked. One of our most important findings was that their mere presence on traditional or social media put researchers at risk for being targeted for online harassment. For these reasons, it is highly important that both service providers and legal officials aim to control the problem.

In our data, online harassment victims were reluctant to report even serious crimes such as death threats to the police because they thought the crime was not serious enough and that reporting it would not lead to any actions. These troubling results indicate that a greater problem of cybercrime is not being recognized as quite as serious as offline offences, despite its clearly negative outcomes both for the victim and society in general. This points to the need for education aimed at increasing understanding of the seriousness of hostile, hateful, and harassing communication online, as well as for a larger discussion considering the possibilities of holding the perpetrators accountable and providing victims with help on an institutional level. The last point is especially important because the authorities’ interventions are often seen as not helpful enough. For instance, a UK study of women involved in online discussions about feminist politics found that only 16% of online harassment victims who reported the offence to the police were satisfied with the intervention’s results (Lewis et al., 2017). This indicates that mere reporting to the police is not enough to combat the problem of hostile online behavior.

Our last implications involve universities as workplaces since the results indicated that online harassment endangers employees’ well-being. Many academics work on short-term contracts and compete for positions and research funding. Both offline and online bullying is a noted problem within academia (DeSouza, 2011). In our study as well, some reported perpetrators of online harassment were from academia. This indicates that online harassment offenders should not be disregarded as merely outsiders. Those victims whose perpetrators came from the university community reported higher PTSD symptoms and lower work engagement, indicating that universities should take the problem very seriously. Furthermore, university staff cannot be passive when facing online harassment. Our results suggest that there are many passive bystanders. Overcoming online harassment faced by university staff will require undertaking actions on many levels. It is not enough for individuals to help only their closest colleagues, as the problem itself concerns everyone. Moreover, explicit procedures on how to report and deal with online harassment are much needed. Along with information on support chains available at the organization, this should include instructions on technical countermeasures offered by social media services. Providing materials on the possibilities of restricting others’ access to an individual’s social media profile and output (e.g., blocking, reporting to site administrators, restricting commenting, and direct messaging) can help scholars feel safe and in control online, allowing them to continue to share their work with the public.

### Limitations and future directions

Despite having the strength of using representative national data of university staff, our study is limited to Finland. However, we believe that the study’s results can be relevant for understanding online harassment in other countries as well. At the minimum, previous cross-national research has indicated similar phenomena in different contexts (Keipi et al., 2017; Reichelmann et al., 2020; Wachs et al., 2021a, b; Zych et al., 2021). The first part of our study was based on cross-sectional data, and future studies should look into the possibilities of using longitudinal data when investigating the potential effects of online harassment. The second part of our analysis had the advantage of using an experimental design, which has not been employed much in previous investigations of online harassment. Our experiment itself was successful, and we were able to confirm part of our preregistered hypotheses. The effect sizes ranged from small to medium, which should be noted.

There is a need for more research on online victimization in the field of higher education studies in general. The problem is growing and global. Cross-national investigations would be an important future avenue. Moreover, there is a need for more studies using an experimental approach to understand how people behave in online harassment situations. Our experiment on reactions to death threats provides an excellent starting point for such studies, but future experimental studies could consider reactions toward other types of online offences. Overall, there is a need for studies using a longitudinal research design in this area. We believe that it is important to find suitable interventions to tackle the phenomenon, and possible interventions should therefore be investigated.

## Conclusions

Online harassment is a growing and persistent problem. Our study, based on nationally representative data of university staff in Finland, revealed that 30% of the participants experienced online harassment during the prior 6 months. Presence in both traditional media and social media were major risk factors for victimization. Victimization was associated with higher psychological distress, lower trust, and lower perceived social support. Victims who were targeted by other members of their university community reported higher PTSD symptoms than other victims. The results of our preregistered experiment showed that participants had stronger reactions to a hypothetical death threat to a close colleague than to an unknown person from the same research field. Overall, our study indicated that the problem of online harassment is highly prevalent. Very few victims reported the assaults to supervisors or police, which is concerning. Also, our study suggested that bystanders of online harassment might be passive online unless the victim belongs to the same in-group. There is an increasing need for organizational, societal, and legislative measures to overcome the problem of online harassment.

## Data Availability

Data is available from the corresponding author with a reasonable request. After the research project, data will be made publicly available in the Finnish Social Science Data Archive (https://www.fsd.tuni.fi/en/).

## Appendix 1

**Table 5:** During the past 6 months, how often have you faced the following situations at work, or because of your work, on the Internet or social media?

Items with exact wording	Yes at least sometimes
You have received offending and angry messages via social media	15.97%
Attacks against you as a person, your values, or your personal life have been made	13.76%
Your appearance has been criticized	4.49%
You have been underestimated or criticized because of your gender	9.79%
You have been underestimated or criticized because of your age	7.14%
You have been attacked because of your sexual orientation	1.52%
You have been attacked because of your skin color, heritage, or national or ethnic origin	5.42%
You have been attacked because of your religion or ideology	6.54%
You have been sexually harassed	5.66%
Your professional skills have been underestimated unjustifiably and beyond normal critique	16.69%
Extracts of your messages have been copied so that the meaning of the original message is distorted	8.47%
Offensive photos/videos of you have been posted on social media	1.04%
Photo or video manipulations of you have been published	0.88%
False statements about you have been spread on social media	8.19%
You have been shamed or targeted (e.g., other people have been provoked to attack you) on social media	4.29%
Someone has impersonated you (identity theft)	1.36%
You have been threatened with violence	2.81%
Your life has been threatened	0.96%
Threatening messages about your friends/family have been sent to you via social media	1.36%
Threatening messages have been sent to your children or close ones with the intention to scare you	0.56%

Response options to each item were:

Never

Sometimes

Monthly

Weekly

Daily

## Appendix 2. Countermeasures asked from the participants of the experiment

1. Block the person on social media.
2. Contact the person personally and ask him/her to stop the harassment.
3. Send a revengeful response.
4. Turn to counseling.
5. Avoid going outside alone when it is dark.
6. Reduce public appearances and participation in public discussions.
7. Change the way he/she talks about his/her work.
8. Think about changing the subject matter of his/her work.
9. Think about transferring to another position or to another field.
10. Report the offence to the police.

## Code availability

Data were analyzed with Stata 16 software. All syntax is available from the corresponding author with a reasonable request.
